# Commentary: Acute effects of cardiac contractility modulation stimulation in conventional 2D and 3D human induced pluripotent stem cell-derived cardiomyocyte models

**DOI:** 10.3389/fphys.2023.1130674

**Published:** 2023-02-09

**Authors:** Mark F. A. Bierhuizen, Jorik H. Amesz, Natasja M. S. De Groot, Yannick J. H. J. Taverne

**Affiliations:** ^1^ Translational Electrophysiology Lab, Lowlands Institute of Bioelectric Medicine, Department of Cardiology, Erasmus University Medical Center, Rotterdam, Netherlands; ^2^ Translational Cardiothoracic Surgery Research Lab, Lowlands Institute of Bioelectric Medicine, Department of Cardiothoracic Surgery, Erasmus University Medical Center, Rotterdam, Netherlands

**Keywords:** living myocardial slices, cardiac contractility modulation (CCM), heart failure, device therapy, engineered cardiac tissue, 3D microphysiological system

With great interest we read the article by Feaster et al. (2022), in which they demonstrated cardiac contractility modulation (CCM) stimulation in 3D human engineered cardiac tissue (ECT). The authors showed that the contractile response of 3D ECT to CCM depends on the input parameters of the stimulation pulse, while this response in conventional 2D pluripotent stem-cell derived cardiomyocytes (hiPSC-CMs) remained unaffected. As such, the authors argue that 3D *in-vitro* models are better suited to evaluate safety and efficacy of novel cardiac devices, including CCM. We want to congratulate the authors, and applaud the initiative to introduce novel pre-clinical human models for medical device testing (Feaster et al., 2022).

CCM stimulation has been investigated in different models and species, but these models were limited by poor *in-vivo* resemblance and lack of extensive experimentation on human tissue. Yet, the exact underlying mechanisms and direct effects of the therapy on human cardiomyocyte physiology remain poorly understood (Brunckhorst et al., 2006). The optimal CCM model should therefore be mechanically loaded, electrically stimulated and of high physiological resemblance. Feaster et al. (2022) greatly contributed to the development of better models to study CCM mechanisms with their 3D ECT. In light of the optimization of such a model, we want to propose human living myocardial slices (LMS) as an additional *in-vitro* platform for CCM testing.

LMS are ultra-thin (300 µm) sections of intact cardiac tissue that maintain structural integrity with intact cellular connections, extracellular matrix proteins and heterocellularity, as they are directly prepared from patient biopsies with a high-precision vibratome (Schneider-Warme et al., 2018; Amesz et al., 2023). LMS are cultured in custom-made biomimetic cultivation chambers at 37°C with near-physiological preload of 1 mN, corresponding to a mean diastolic wall stress of 0.66 kN/m^2^ (Fischer et al., 2019; Amesz et al., 2023). Electrical stimulation is established with graphite field electrodes, leading to cardiac contraction of the LMS (Fischer et al., 2019; Amesz et al., 2023). In comparison to 3D-ECT, LMS represent more accurate *in-vivo* mimicry, because the complex microarchitecture of the cardiac system including all cell types and extracellular matrix proteins is difficult to mimic *in-vitro*, and hiPSC-CMs often fail to show complete cardiac maturity (Qu et al., 2020). Moreover, LMS can be produced from patients with end-stage HF, enabling the possibility to study CCM in the tissue of the population it was intended for.

Programmed CCM stimulation can be established via the electrodes of biomimetic cultivation chambers as a second pulse during the refractory period and dedicated force transducers continuously measure differences in contractility of the LMS. In addition, LMS of patients with HF remain beating for several months enabling studies on the chronic effects of CCM on cardiac contractility (Fischer et al., 2019). Furthermore, the LMS platform also contains ample opportunities for additional analyses to unravel the mechanisms of action of CCM, including culture medium biochemistry, histology of LMS and electrophysiology (Figure 1).

**FIGURE 1:**
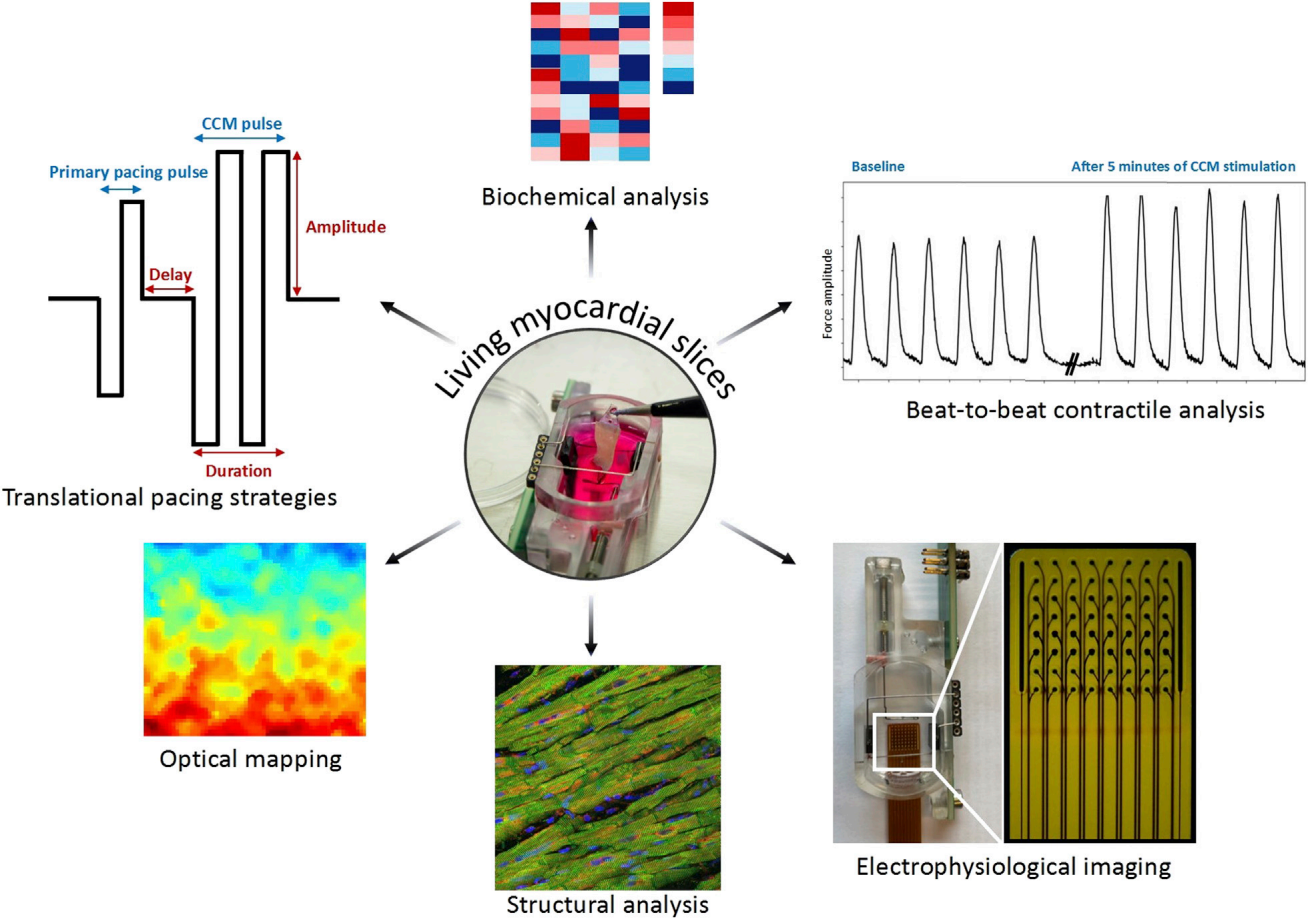
Living myocardial slices form a representative human *in vitro* translational cardiac device research platform.

In conclusion, Feaster et al. (2022) showed an important novel model for CCM studies supporting our belief that pre-clinical CCM testing in human tissue is necessary for better understanding of underlying CCM mechanisms. LMS form an additional, representative human *in vitro* platform and might accelerate this journey towards translational CCM and other cardiac devices research.

## References

[B1] Amesz J. H. Zhang L. Everts B. R. De Groot N. Taverne Y. J. H. J. (2023). Living myocardial slices: Advancing arrhythmia research. Front. Physiology 14, 17. 10.3389/fphys.2023.1076261 PMC9880234 36711023

[B2] Brunckhorst C. B. Shemer I. Mika Y. Ben-Haim S. A. Burkhoff D. (2006). Cardiac contractility modulation by non-excitatory currents: Studies in isolated cardiac muscle. Eur. J. Heart Fail 8, 7–15. 10.1016/j.ejheart.2005.05.011 16202650

[B3] Feaster T. K. Feric N. Pallotta I. Narkar A. Casciola M. Graziano M. P. (2022). Acute effects of cardiac contractility modulation stimulation in conventional 2D and 3D human induced pluripotent stem cell-derived cardiomyocyte models. Front. Physiol. 13, 1023563. 10.3389/fphys.2022.1023563 36439258 PMC9686332

[B4] Fischer C. Milting H. Fein E. Reiser E. Lu K. Seidel T. (2019). Long-term functional and structural preservation of precision-cut human myocardium under continuous electromechanical stimulation *in vitro* . Nat. Commun. 10, 117. 10.1038/s41467-018-08003-1 30631059 PMC6328583

[B5] Qu Y. Feric N. Pallotta I. Singh R. Sobbi R. Vargas H. M. (2020). Inotropic assessment in engineered 3D cardiac tissues using human induced pluripotent stem cell-derived cardiomyocytes in the Biowire(TM) II platform. J. Pharmacol. Toxicol. Methods 105, 106886. 10.1016/j.vascn.2020.106886 32629159

[B6] Schneider-Warme F. Johnston C. M. Kohl P. (2018). Organotypic myocardial slices as model system to study heterocellular interactions. Cardiovasc Res. 114, 3–6. 10.1093/cvr/cvx215 29121179

